# Voltage and pH sensing by the voltage-gated proton channel, H_V_1

**DOI:** 10.1098/rsif.2018.0108

**Published:** 2018-04-11

**Authors:** Thomas E. DeCoursey

**Affiliations:** Department of Physiology & Biophysics, Rush University, 1750 West Harrison, Chicago, IL 60612, USA

**Keywords:** ion channels, pH, proton conduction, voltage gating, proton transport

## Abstract

Voltage-gated proton channels are unique ion channels, membrane proteins that allow protons but no other ions to cross cell membranes. They are found in diverse species, from unicellular marine life to humans. In all cells, their function requires that they open and conduct current only under certain conditions, typically when the electrochemical gradient for protons is outwards. Consequently, these proteins behave like rectifiers, conducting protons out of cells. Their activity has electrical consequences and also changes the pH on both sides of the membrane. Here we summarize what is known about the way these proteins sense the membrane potential and the pH inside and outside the cell. Currently, it is hypothesized that membrane potential is sensed by permanently charged arginines (with very high p*K*_a_) within the protein, which results in parts of the protein moving to produce a conduction pathway. The mechanism of pH sensing appears to involve titratable side chains of particular amino acids. For this purpose their p*K*_a_ needs to be within the operational pH range. We propose a ‘counter-charge’ model for pH sensing in which electrostatic interactions within the protein are selectively disrupted by protonation of internally or externally accessible groups.

## 1. Introduction

The focus of this review is how the voltage-gated proton channel, H_V_1, senses voltage and pH. H_V_1 is a unique ion channel, a membrane protein that allows protons (H^+^) but no other ions to cross cell membranes. Its existence was first postulated in 1972 by Hastings and co-workers [1], who proposed that it triggered the flash in bioluminescent dinoflagellates, a role that was recently confirmed [2,3]. Proof of the existence of H_V_1 was produced a decade later by Thomas and Meech with their 1982 voltage-clamp study of snail neurons [4]. Nearly a quarter of a century later, the gene for voltage-gated proton channels was finally identified [5,6]. Subsequently, proton currents have been identified in cells from 15 species, and HVCN1 genes (that code for H_V_1) in another 11 species have been confirmed by expression in heterologous systems and voltage clamp. To date, only one gene per species has been found, although, in several cases, truncated isoforms have been identified [7–9]. An astonishing variety of functions have been identified in these phylogenetically disparate species, many of which are listed in table 1. Involvement of H_V_1 in human health is extensive [48], but beyond the scope of this review.

**Table 1.** Types of functions proposed for H_V_1 in different cells. Functions proposed for H_V_1 in various cells are sorted into the four main effects of H_V_1 activity, in some cases arbitrarily. NOX is NADPH oxidase. BCR is B cell receptor. *V*_m_ is membrane potential.

cell	↑pH_i_	↓pH_o_	regulate *V*_m_	charge compensation
dinoflagellates	trigger bioluminescent flash [1,3]; feeding [2]		proton action potential [3]	
coccolithophores	calcification [10]			
insect	acid extrusion [11]			
snail neurons	acid extrusion [4,12,13]	acidification of confined spaces [14]		ROS production for host defence [15]
amphibian oocyte	maturation, fertilization [16,17]		*V*_m_ oscillations [18]	
zebra fish				neutrophils [19]
respiratory epithelium	acid extrusion [20]	optimize pH of airway surface fluid [21]; CO_2_ extrusion [22]		facilitate DUOX1 activity [23]
skeletal myotubes	acid extrusion [24]			
phagocyte	optimize pH_i_ for NOX [25–27]	phagosome pH and volume [28,29]	regulate *V*_m_ [28,30]; avoid apoptosis [31]	prevent NOX self-inhibition at high potentials [32–34]
microglia	optimize pH_i_ for NOX [35]; volume regulation [36]			ROS production [35,37]
basophil	histamine secretion [38]			
cardiac myocytes		CO_2_ elimination [39]		
cardiac fibroblasts			regulate *V*_m_ [40]	
osteoclasts	acid extrusion [41]		regulate *V*_m_ [41]	
sperm	alkaline pH_i_ triggers capacitation [42]			ROS production by NOX5 mediates motility [43]
cancer cells	tumour growth [44]	metastasis [44]		
B lymphocyte				ROS production in BCR signalling [7,45]
malignant B cells				short isoform promotes proliferation [8]
kidney	acid extrusion [46]			Na^+^-dependent ROS production [47]

The protein at the focus of this chapter is the voltage-gated proton channel, H_V_1. Being ‘voltage-gated’ means that it can sense voltage, specifically the electrical potential difference across a cell membrane. As indicated by its name, the voltage-gated proton channel is an ion channel that conducts protons selectively when it is opened by depolarizing transmembrane voltages (making the membrane potential—the difference in voltage inside the cell compared with outside—more positive). H_V_1 channels open in response to depolarization, and they close with hyperpolarization (more negative membrane potentials). How this occurs will be discussed. A crucial and unique property of the H_V_1 channel is that its voltage sensitivity is modulated profoundly by the pH. Therefore, a second focus of this review is how the protein senses and responds to the pH. The aim of this review is to describe the current state of understanding of the gating mechanism of H_V_1. Gating is a quintessential property of all ion channels—a channel without gating is simply a pernicious shunt that would rapidly dissipate the membrane potential as well as the concentration gradient of any ions to which it is permeable. Ion gradients are required to drive transport of substances into or out of the cell, to generate energy from food and to conduct electrical impulses in excitable cells. By ‘gating’ we mean that the channel exists in at least two distinct functional states, ‘closed’ and ‘open’. When the channel is closed, it does not conduct current. To be precise, one K^+^ channel was shown to conduct detectably when closed, but the ‘closed’ channel current was more than 10^5^ smaller current than the open channel current [49]. When a channel is open, it conducts current in the form of ions, usually at a constant rate. Ion channels differ from other transporters in being completely passive, conducting ions according to their electrochemical gradient. The chemical gradient drives ions from the side with higher concentration towards the side with lower concentration. The size and direction of ionic current is also sensitive to the electrical potential across the membrane, which can drive current in either direction, either supported or opposed by the chemical gradient. The membrane potential at which the electrical and concentration gradients balance is the Nernst potential [50]. For example, the Nernst potential for H^+^ (*E*_H_) is
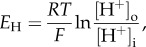
where *R* is the gas constant, *T* is the absolute temperature, *F* is Faraday's constant, [H^+^] is the proton concentration, and the subscripts o and i mean outside and inside the cell, respectively. If the membrane potential is positive to *E*_H,_ protons will be driven out of the cell; at voltages negative to *E*_H,_ they will enter the cell. The Nernst potential is useful experimentally to establish the ion selectivity of a channel. Current through a proton-selective channel will reverse at *E*_H_ regardless of the presence of other ions.

**Figure 1.**
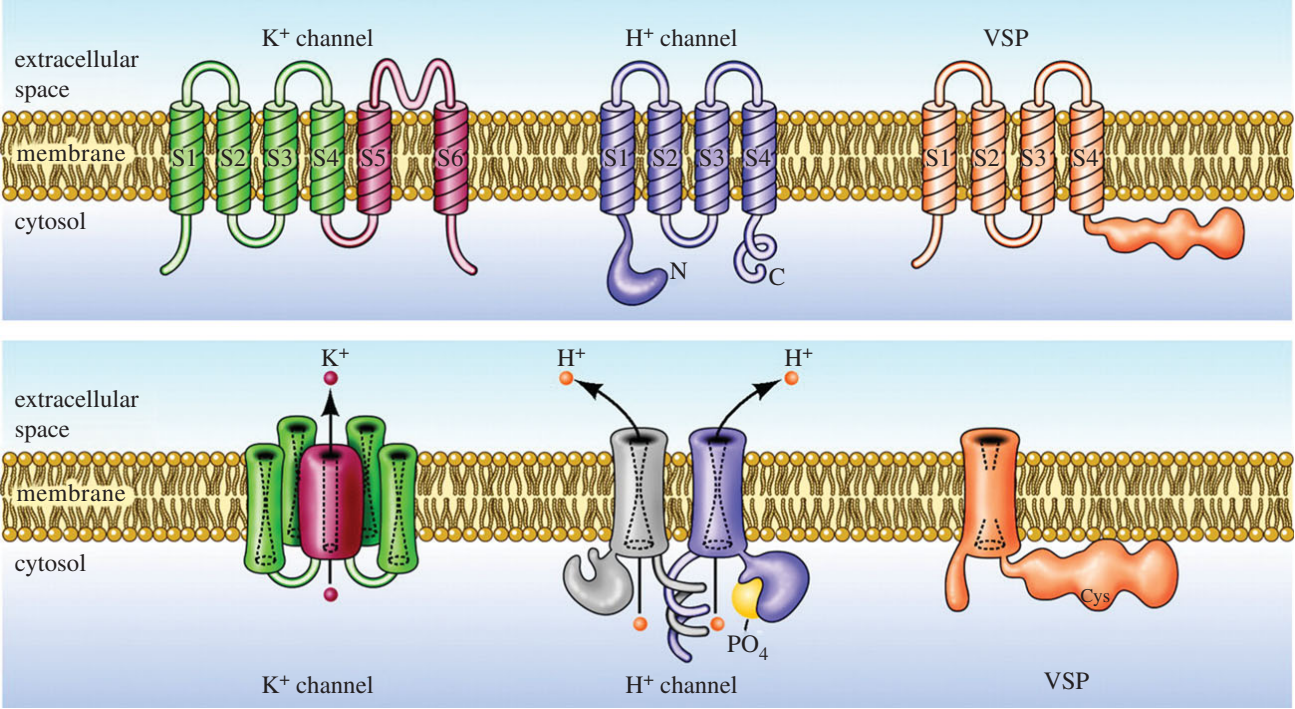
Architectural features of voltage-gated K^+^ channels, H_V_1 channels and voltage-sensing phosphatases (VSP). The top row shows monomeric subunits of the complete molecule in the lower row. K^+^ channels are homotetramers with six transmembrane helices per monomer. Segments S1–S4 form the voltage-sensing domain (VSD) and S5–S6 form the conduction pathway. In the complete assembled channel (below), four VSDs (each comprising S1–S4) surround a single central pore through which K^+^ permeates. Dashed lines indicate central aqueous regions inside each VSD. H_V_1 resembles an isolated VSD with only four TM segments and no explicit pore domain [5,6], but functions without accessory proteins [51]. It forms a dimer, largely due to coiled-coil interaction in the C terminus, but each protomer has its own conduction pathway [52–54]. Phosphorylation of Thr^29^ in the N terminus [8,55] greatly enhances H_V_1 activity [56], especially in phagocytes [57]. The VSP lacks conduction altogether, but senses voltage and modulates phosphatatse activity accordingly [58,59]. Reprinted with permission from DeCoursey [60] (Copyright © 2010 American Physiological Society).

The topology of H_V_1 is illustrated by the cartoon in figure 1, which emphasizes the similarities and differences between molecules that contain voltage-sensing domains (VSDs). The VSD contains a number of charged amino acids that are thought to move when changes in membrane potential alter the electric field within the membrane. Most of these charges are Arg, or occasionally Lys, which are located every third position in the S4 helix and are thought to face the pore [61]. Voltage-gated K^+^ and Na^+^ channels have up to seven charged groups in S4, the VSP has four and H_V_1 has only three. In K^+^ channels, applied voltage causes the four VSDs to move and pull on parts of the central pore domain (S5 and S6), causing an opening to appear through which K^+^ as well as water molecules can pass. Ions are hydrophilic and can diffuse through water rapidly, but they avoid entering hydrophobic regions of cell membranes or of membrane proteins. Most ion channels are thought to have narrow hydrophilic regions where ions and water must move in single file. The conduction pathway of H_V_1 is within its VSD, which comprises the entire transmembrane region (S1–S4), whereas the VSDs in other membrane proteins serve mainly to sense voltage, and then in response they open a separate pore (e.g. the K^+^ channel) or turn-on an enzyme (e.g. the voltage-sensing phosphatase, VSP). As indicated schematically by the dashed hourglass shapes in figure 1, each VSD contains aqueous vestibules with a narrow constriction at the middle of the membrane. The constriction in H_V_1 conducts protons, but in K^+^ channels or the VSP they normally do not conduct at all. Intriguingly, if the K^+^ pore domain is removed altogether, the K^+^ channel VSD in isolation forms a proton-conducting channel [62]. Even when the molecules are still intact and attached to their pore domains, the VSDs of both voltage-gated K^+^ [63–65] and Na^+^ channels [66,67] can be induced to conduct protons by mutating particular Arg in the S4 segment to histidine (His). This mutation would result in four proton-conducting VSDs surrounding the central K^+^-conducting pore. His are well known for their ability to transfer protons [68,69], as they do in the M2 influenza A viral proton channel [70] and in carbonic anhydrase II [71]. Similarly, when Arg^205^ (the first—outermost—of three Arg (arginines) in S4)^1^ in hH_V_1 is replaced by His, inward proton current is detectable [72]. All of these ‘gating pore’ currents support the idea that the VSD resembles an hourglass with aqueous vestibules separated by a narrow hydrophobic region. In the guise of hydronium ions, protons can reach most places that water can. Although aquaporin channels normally conduct water at a high rate but exclude protons, showing that this is not a firm rule, point mutations can enable proton conduction even through aquaporin [73,74]. Presumably, a single His at the centre of a VSD can, perhaps with a bit of wiggling around, access both external and internal solutions and transfer a proton across this narrow bridge. This is what H_V_1 normally does whenever it opens, except without the benefit of His. In hH_V_1, the proton is transferred by the carboxyl group of Asp^112^ [75,76] and perhaps other acidic groups [77], as occurs in numerous proton pathways in other pumps and enzymes [69,78–81].

We will identify H_V_1 from different species with prefixes, hH_V_1 = human, mH_V_1 = mouse, otherwise two letters for genus and species, e.g. CiH_V_1 = *Ciona intestinalis*. Although there are some apparent differences [82], the functional similarities among H_V_1 from widely disparate species are remarkable.

## 2. H_V_1 exhibits cooperative gating

**Figure 2.**
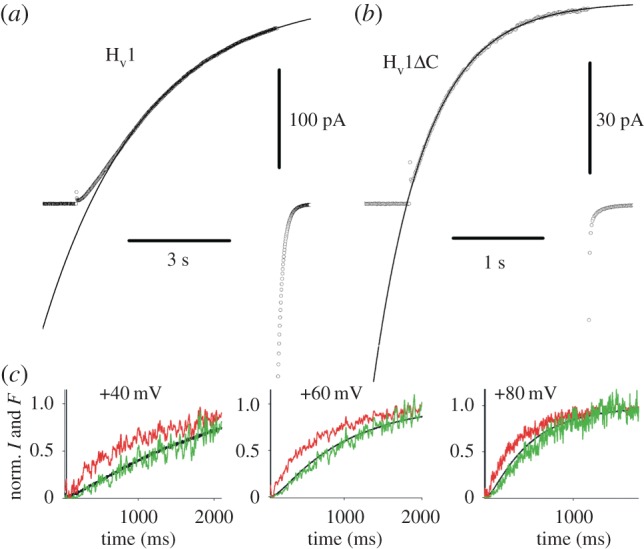
Cooperative gating of H_V_1. The H_V_1 dimer behaves as expected of a classical Hodgkin–Huxley *n*^2^ system. The WT channel in most species is a dimer in which both protomers must undergo a conformational change before either conducts. This manifests as sigmoidal activation kinetics in hH_V_1 (*a*). Truncation of the C terminus eliminates coiled-coil interaction and results in each monomer apparently functioning independently. In monomeric constructs (*b*), activation is exponential and is five to seven times faster than in the dimer [52,83,84]. Lines show single exponential fits; both currents were recorded at +50 mV at a symmetrical pH of 7.5. In (*c*), the red trace is the fluorescence signal from a tag attached to S4 in dimeric CiH_V_1, showing the exponential time course of its movement. The black trace shows the current, with its sigmoid turn-on. The green trace is the square of the red fluorescence signal, matching the current in the classical Hodgkin–Huxley manner, in which both protomers must activate before current is observed. (*a*,*b*) Reprinted with permission from Musset *et al.* [83] (Copyright © 2010 The Physiological Society) and (*c*) Reprinted with permission from Gonzalez *et al.* [85] (Copyright © 2010 Nature Publishing Group).

As shown in figure 1, H_V_1 is a dimer, but each protomer has its own conduction pathway. The channel is voltage-gated, meaning that it opens when the membrane potential is depolarized, i.e. made more positive. Compared to most voltage-gated ion channels, H_V_1 opens extremely slowly (figure 2), at least in mammals. Voltage-gated Na^+^ channels, for example, open within a millisecond or so, triggering an action potential in nerve or muscle cells. K^+^ channels in the same cells open only slightly more slowly, to repolarize the membrane. Although in snail neurons where proton currents were first identified, H_V_1 are as fast as other channels [4,12], mammalian H_V_1 are approximately 10^3^ slower. When H_V_1 is forced to function as a monomer, it continues to exhibit the main properties of the dimer, but it opens five to seven times faster [52,83,84], as seen in figures 2*a,b*.

There is agreement that the two protomers in the H_V_1 dimer gate cooperatively, but not on precisely what ‘cooperative gating’ means. One definition of cooperative gating of H_V_1 is that a voltage-sensitive conformational change (generally envisaged as outward movement of the S4 helix) must occur in each protomer before either one can conduct [85]. Figure 2*c* illustrates that H_V_1 is well described by this type of cooperative gating, analogous to that proposed by Hodgkin and Huxley for squid axon Na^+^ and K^+^ channels [86]. In the latter channels, three to four identical ‘particles’ (what we now would call VSDs) were proposed, and molecular biology eventually confirmed that there are four subunits in both types of (tetrameric) channel [87]. An alternative proposal is that strong positive cooperativity exists in H_V_1 such that opening of one protomer greatly accelerates the opening of the other [88], analogous to the cooperative binding of oxygen to the four haem groups in a haemoglobin molecule. The gating model discussed below in §5.2 illustrates a possible mechanism for ΔpH dependent gating, which will be discussed later. In this model, the channel gates cooperatively, and the final concerted opening process results from protonation of both protomers at internal locations [89].

## 3. What is the difference between open and closed H_V_1 channels?

A straightforward, if reductionistic, way to dissect the physical process of gating would be to compare the structures of open and closed H_V_1 channels, which would reveal which parts must move and by how much. This is not possible at present, because only one crystal structure exists and this is presumed to be in a closed state (i.e. the membrane potential in the crystal is effectively 0 mV and mH_V_1 is closed at 0 mV). The molecule crystallized was a chimeric protein that includes parts of mouse H_V_1 (mH_V_1) spliced together with parts of two other proteins [90]. In addition, electron paramagnetic resonance (EPR) spectroscopy data exist for the human H_V_1, hH_V_1, also in a presumed closed state [91]. A growing number of homology models have been produced that often reflect the preconceived notions of their creators [72,77,83,92–98]. These are based mostly on homology with the VSDs of other voltage-gated ion channels [99]. The main differences are with regard to the extent of movement of the S4 helix during gating. A different approach by Li *et al*. [91] was to use the crystal structures of the ‘down’ and ‘up’ states of a voltage-sensing phosphatase [100] as templates. To create a homology model, a starting conformation is selected based on structures of homologous proteins, which is allowed to relax using molecular dynamics (MD) simulations. Sometimes multiple possible templates are assumed and statistical analysis or other criteria reveal the more probable model [93,94,99]. Accurate homology models would provide a starting point for understanding gating. However, the value of structural information, preferably structures for both closed and open channel proteins, cannot be overstated.

The question how open and closed channels differ is more difficult and subtle for H_V_1 than it is for other kinds of ion channels, because protons can and do traverse pathways that other ions cannot [68,69,101–107]. Normal ions require a pore wide enough to accommodate them, typically accompanied by water; often there is a ‘single-file’ region within which ions and water molecules cannot pass each other [87,108–110]. Proton pathways through proteins consist of hydrogen-bonded chains that may include any combination of water and side chains of certain amino acids [68,78,80,101,111–113]. Protons transfer across hydrogen bonds between waters or titratable groups; exclusion of other ions can be achieved by packing the protein to preclude water or foreign ion permeation [69]. There need not be any ‘pore’ as such; in principle, a channel might conduct protons but not water. In contrast with other ion channels, it is not obvious *a priori* that conducting and non-conducting states of proton channels would differ in any predictable and easily observable way. For example, the M2 proton channel of influenza A virus ‘opens’ to conduct H^+^ with high [114,115], albeit not perfect, selectivity [116–118] when the third of four His residues in a ring is protonated [119–124]; this produces only a subtle conformational change—a slight expansion of the ring due to electrostatic repulsion, which is nevertheless sufficient to permit H^+^ shuttling.

Protons (and to a lesser extent OH^–^) diffuse through water approximately five times faster than other ions [125] because the proton alone can move by a Grotthuss hopping mechanism [126,127]. Other ions must diffuse around waters, whereas protons can save time and distance by virtually hopping ‘through’ waters because, crucially, the identity of the proton may change with each transfer [128]. Water contains 110 M hydrogen atoms, each of which is interchangeable with the excess proton in H_3_O^+^. Because the mechanism of proton transfer in water is highly efficient, proton pathways through proteins typically comprise mostly water [103].

The crystal structure of closed H_V_1 revealed two hydrophobic regions in the permeation pathway [90]. The presumption was that these hydrophobic zones would be smaller or absent in the open (conducting) state. However, most homology models of the open state of H_V_1 also predict distinctly hydrophobic regions in the H_V_1 permeation pathway [94,96,98]. It is instructive that the mean hydration profile calculated for a series of hH_V_1 mutants was indistinguishable for constructs found experimentally to be proton-selective, anion-permeable or non-conducting [95]. In addition, an MD simulation of presumed closed and open states of hH_V_1 revealed no clear wet/dry transitions [98]. Nevertheless, the conductance of hH_V_1 mutants in which Asp at position 112 was replaced with various neutral amino acids decreased with hydrophobicity of the substituent, to zero (undetectable) for the two most hydrophobic amino acids Val and Ile (fig. 9 in [129]). Evidently, a sufficiently hydrophobic region can occlude even H^+^ conduction, but whether H_V_1 gating uses a tunable hydrophobic constriction [96] is not clear at present. Introduction of His into the S4 helix as R205H results in proton leakage in closed hH_V_1 channels [72]. This result suggests that H^+^ conduction is occluded at only a single constriction in the closed state.

## 4. What evidence supports molecular movement during gating of H_V_1 channels?

### 4.1. What evidence supports molecular movement during gating of other voltage-gated ion channels?

In part because of its relative novelty, most people who study H_V_1 today previously or contemporaneously worked on other ion channels. Consequently, we all have preconceptions of how voltage-gated channels work, and we tend to project onto H_V_1 the properties and mechanisms that apply to other (more extensively studied) channels. Several types of experimental evidence provide information about the extent of molecular movement of channels: gating currents, accessibility studies, FRET (fluorescence resonance energy transfer) measurements, and structural studies including X-ray crystallography and EPR. Similar experiments performed on H_V_1 are discussed in § 4.3. Most such studies indicate substantial movement of the S4 transmembrane segment during gating of other voltage-gated ion channels [110,130–139]. The S4 helix is believed to be the main voltage-sensing element of voltage-gated ion channels, because it has a series of cationic residues (mostly Arg with occasionally Lys) along its inner wall, spaced at every third position so that they line the pore [61,140]. It is widely believed that voltage gating occurs when S4 moves outwards, with a twist [141]. Recent studies conclude that four Arg move from intracellular towards extracellular positions in the *Shaker* K^+^ channel [134,142–145]. Because the cationic Arg are thought to interact with acidic residues in other parts of the VSD [146–147], one may quantify S4 movement by the number of discrete ‘clicks’ each Arg moves, being stabilized sequentially by the negatively charged groups as S4 ratchets outwards. Tao *et al*. [143] proposed that each gating charge moves through a ‘gating charge transfer center’ where it interacts with two acidic groups. S4 appears to move slightly less in Na^+^ channels [148,149], and even less in CiVSP, just one click [100]. The default starting point of our imagination is, therefore, our view of how other VSD-containing molecules move during gating.

We now step back to the foundation of modern ion channel research, Hodgkin & Huxley [86]. Based on their pioneering application of the voltage-clamp technique, they proposed that the pathway for ionic currents could be activated by the movement of a large quantity of charge across the membrane. They measured ionic currents using voltage clamp, and from the maximum current (*I*) at each voltage (*V*) and Ohm's Law they calculated the conductance (inversely related to resistance) *G* = 1/*R* = *I*/*V*. Assuming that current through a single type of channel has been isolated, the conductance is roughly proportional to the fraction of channels that open at each voltage. The *G*–*V* relationship thus shows the probability of channel opening as a function of voltage. Hodgkin and Huxley commented on the extreme steepness of the *G*–*V* relationship, from which they calculated that the equivalent of six elementary charges (*e*_0_) must cross the membrane for each conduction site (now called a ‘channel’). Later, more sophisticated estimates increased the gating charge for voltage-gated Na^+^, K^+^ and even Ca^2+^ channels to 12–14 *e*_0_ per channel [150–154]. The gating currents predicted by Hodgkin and Huxley to reflect the movement of charges within the membrane have been detected [155–158]. A crucial discovery was that replacing each of four Arg in S4 individually with His produced a proton-selective pathway through the *Shaker* K^+^ channel VSD [63–65]. Each mutant behaved as though protons (carried on a hydronium ion) could approach the His, bind, and then be translocated to the other side. This provided strong evidence that only a quite narrow region of the VSD is inaccessible to aqueous solution; and that the VSD is hourglass-shaped with large aqueous vestibules. If the first Arg is replaced by an amino acid smaller than His, a non-selective cation current is seen [159]. Intriguingly, even without Arg mutation, the isolated *Shaker* VSD (i.e. with the pore domain S5–S6 removed) conducts cations, with a strong preference for protons [62]. The short region between the vestibules has been called variously a hydrophobic gasket [91,160], hydrophobic plug [62,96,161,162,] or hydrophobic barrier [163]. The importance for voltage gating is that most of the transmembrane electric field drops across this hydrophobic region. The aqueous vestibules are low-resistance pathways in series with the high-resistance hydrophobic gasket. Consequently, if a charge moves or switches its accessibility from one side of the short hydrophobic region to the other, the result is electrically indistinguishable from the charge crossing the entire membrane.

### 4.2. Gating charge movement

Gating mechanisms can be constrained by measuring the amount of charge that moves when the channel opens, as ‘gating current’. Unfortunately, this measurement is more difficult for H_V_1 than for other channels [82]. Direct measurement of gating currents using voltage clamp requires eliminating the permeant ion, which is impossible for H^+^, or blocking the current by occlusion, but all known potent inhibitors of H_V_1 modify gating and exhibit state dependence [164,165]. A recent approach is to measure H_V_1 gating currents in a non-conducting mutant [166,167]. Very rough estimates of gating charge can be obtained from the slope of a Boltzmann function fit to the *g*_H_–*V* relationship; a more reliable estimate can be obtained from its limiting slope at large negative voltages [168,169].

The channel at the focus of this review, H_V_1, has only three Arg in its S4 helix [5,6], which remains true for confirmed H_V_1 in all species thus far [2,3,10,11,19,170–172]. It is, however, a dimer that operates cooperatively [52–54,83,85,88,173,174]. If all three Arg moved effectively across the entire membrane electical field when H_V_1 opened, one would predict a gating charge of 6 *e*_0_ for the dimer. Remarkably, this is precisely the value that was obtained from limiting slope measurements a decade before the gene was identified [175,176]! Tetrameric voltage-gated ion channels have four VSDs (figure 1), each moving approximately three charges for a total of 12–14 *e*_0_; the two VSDs of the H_V_1 dimer together move half this charge. Similar values have been measured in heterologously expressed proton channels: 6 *e*_0_ for hH_V_1, [177], 6 *e*_0_ for CiHv1 [178], 5.5 for HtH_V_1 [172] and 4 *e*_0_ for mH_V_1 [84]. Consistent with the cooperative gating mechanism, monomeric constructs exhibit gating charge just half of those values: 3 *e*_0_ for CiHv1 [85] and 2 *e*_0_ for mH_V_1 [84]. Finally, mutation of each of the three Arg in S4 to Asn reduced the gating charge assessed by the limiting slope method [178]. However, despite everything working out so neatly, it is not clear that all of the gating charge movement in H_V_1 results from S4 movement (as will be seen shortly.

### 4.3. Accessibility of various parts of the H_V_1 protein to aqueous solution

Membrane proteins are proteins embedded in the plasma or organelle membranes of cells. The accessibility of specific locations on a protein gives clues to its gross topology. The parts of the protein that are in contact with the aqueous solutions on either side of the membrane should be accessible to water-soluble probe molecules. Sites buried within the protein or that abut the membrane are not likely to be accessible. In a commonly used technique called ‘cysteine scanning mutagenesis' or ‘Cys scanning’, individual amino acids are replaced with Cys, and then probed with MTS (methanethiosulfonate) reagents [133,135]. If a Cys is accessible, MTS reagents may react with it and alter channel function. Under voltage clamp, the sidedness and state dependence (i.e. whether accessibility differs when the channel is open or closed) of MTS action can be determined. The ‘PEGylation protection’ assay also uses Cys scanning, but requires western blots which cannot be done *in vivo* and thus reveals accessibility only of presumed closed channels (because there is no membrane potential) and does not distinguish sidedness [179–181]. Cys scanning and MTS modification of CiH_V_1 channels in open or closed states clearly show changes in accessibility consistent with outward S4 movement of roughly one click [85,178]. Accessibility changes in the S1 segment are consistent with inward movement of S1 or simply widening of the internal vestibule of hH_V_1 [182]. Inward movement of S1, which has two to three negatively charged groups (Asp and Glu), and outward movement of S4, with its three cationic Arg, could both contribute to measured gating charge movement.

**Figure 3.**
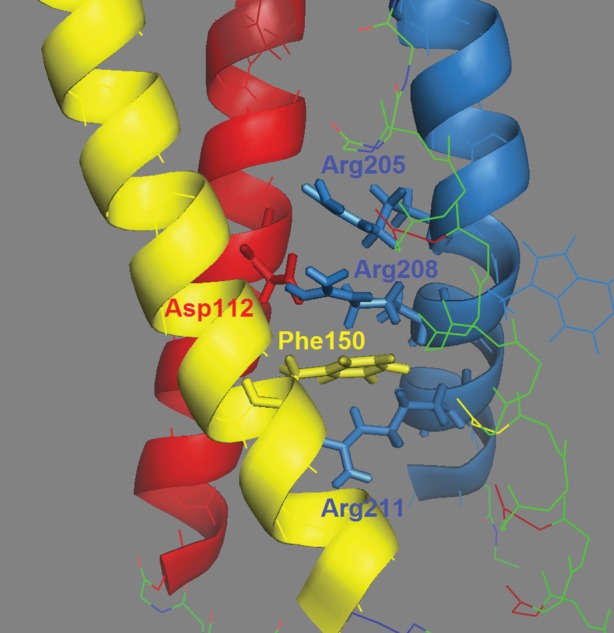
Side view of the open human H_V_1 channel, with the external end up. Transmembrane helices are colour-coded: S1 = red, S2 = yellow, S4 = blue and S3 is shown as lines to be unobtrusive. Key amino acids are labelled and shown with side chains as sticks. Asp^112^ is crucial for selectivity; Phe^150^ demarcates inner and outer aqueous vestibules and the three Arg in S4 sense voltage. Figure is based on the model of Li *et al.* [91]. Note that Asp^112^ interacts with Asp^208^ [94], and Arg^211^ is below Phe^150^, and thus is exposed to the inner vestibule. Drawn with PyMol.

Accessibility of specific locations in the protein can be assessed in other ways. Introducing a pair of Cys or His residues and then probing with metals (Cd^2+^ or Zn^2+^) under voltage clamp can reveal state-dependent interactions (i.e. the metal binds preferentially in open or closed channels) [183,184]. When the three Arg in S4 of hH_V_1 (figure 3) were individually replaced with His and probed with Zn^2+^ in the open state, the outermost two, R1 and R2, were accessible to the external solution, but R3, Arg^211^ was not. R2 appeared also to be internally accessible, presumably in closed channels, but the innermost Arg, R3, was accessible only from the internal solution and was clearly accessible even in the open state [94,95]. These data were interpreted as indicating that a one-click outward movement of S4 was sufficient to result in hH_V_1 opening. In the closed crystal structure of H_V_1 [90], the Asp^112^ in the middle of S1 that is crucial to proton selectivity [75] interacts with the first Arg in S4 (R1 or Arg^205^). In our model, Asp^112^ interacts with the second Arg of S4, Arg^208^ in the open channel [94,95]. Statistical analysis of extensive MD simulations of the open hH_V_1 model to compare the assumptions that Asp^112^ interacted either with R2 or R3 consistently supported the stability of the R2 interaction [94]. If the R211H mutation can be taken at face value, models in which the third Arg moves all the way into the external vestibule must be ruled out. As with all mutations, the interpretation of R211H assumes that the molecule behaves essentially identically to wild-type (WT). It is also evident that neutralizing the cationic Arg in S4 may alter the extent of S4 movement [178]. His is a conditionally conservative replacement for Arg in that it might be cationic, but its p*K*_a_ in solution is 6.5 and this could be altered by the local environment within the protein, and thus its protonation state even at pH 6.0 is not clear. Another note of caution is that the hH_V_1 molecule is highly dynamic, even more than other VSDs [91], and this mobility might manifest as the molecule sampling a wide range of conformations. Thus accessibility by any criterion will have a statistical component. However, that R3 is not externally accessible in spite of the high molecular mobility strengthens the argument that S4 outward movement is limited.

## 5. What is the mechanism of ΔpH-dependent gating?

### 5.1. What is ΔpH-dependent gating?

**Figure 4.**
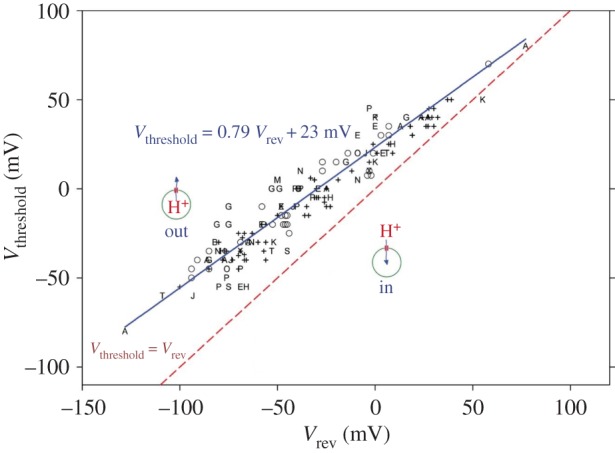
The ΔpH dependence of gating ensures that, in most species, H_V_1 channels open only when doing so will result in acid extrusion. Each symbol or letter indicates a different cell type or species, defined in [68]. The blue line shows the relationship that results in H^+^ channels in almost all cells opening at their threshold voltage, *V*_threshold_, at voltages positive to the Nernst potential (or the experimentally measured reversal potential, *V*_rev_), shown as the red dashed line. At more positive voltages (above the blue line) more channels would open, extruding more protons. Reprinted with permission from DeCoursey & Hosler [69], with modification (Copyright © 2014 Rockefeller University Press).

One of the most distinctive properties of H_V_1 is ΔpH-dependent gating [89]. This feature occurs universally in all species studied thus far and is essential to all of its functions [48]. The biological significance of ΔpH dependence is that H_V_1 acts to extrude acid from cells (figure 4). The channel is regulated by pH so that (with rare exceptions) it only opens when doing so will result in outward H^+^ current. This functional ‘rectification’ is due almost entirely to the pH dependence of gating, and does not reflect rectification of the open channel current. Under symmetrical pH conditions (pH_o_ = pH_i_) the open H_V_1 channel conducts outward current somewhat better than inward, but by a factor of less than 2 [89]. Four types of consequences of H_V_1 activity can be listed (table 1), although some proposed functions do not fit neatly into these categories or have uncertain mechanisms. H^+^ efflux will change pH on both sides of the membrane, depending on the situation, pH_o_ or pH_i_ may be more critical. One could subdivide these further: in the face of an acid load, H^+^ efflux serves to keep pH_i_ constant, but increasing pH_i_ is a signal for sperm capacitation [42]. In a number of cells, the electrical consequences of H_V_1 activity are crucial (table 1). The best studied example is charge compensation during the phagocyte ‘respiratory burst’, i.e. NADPH oxidase (NOX) activity. NOX is electrogenic [32,34,57,185] and produces massive depolarization in neutrophils [186–188]. H_V_1 compensates for the electron efflux through NOX, limiting the extent of depolarization [28,30,33,34,189]. Without H_V_1, the NOX-induced depolarization would rapidly produce self-inhibition [28,33,34]. Another cell that uses the electrical manifestations of H_V_1 activity is the dinoflagellate, in which an H_V_1-mediated action potential triggers the bioluminescent flash [2,3].

### 5.2. How does ΔpH-dependent gating work?

**Figure 5.**
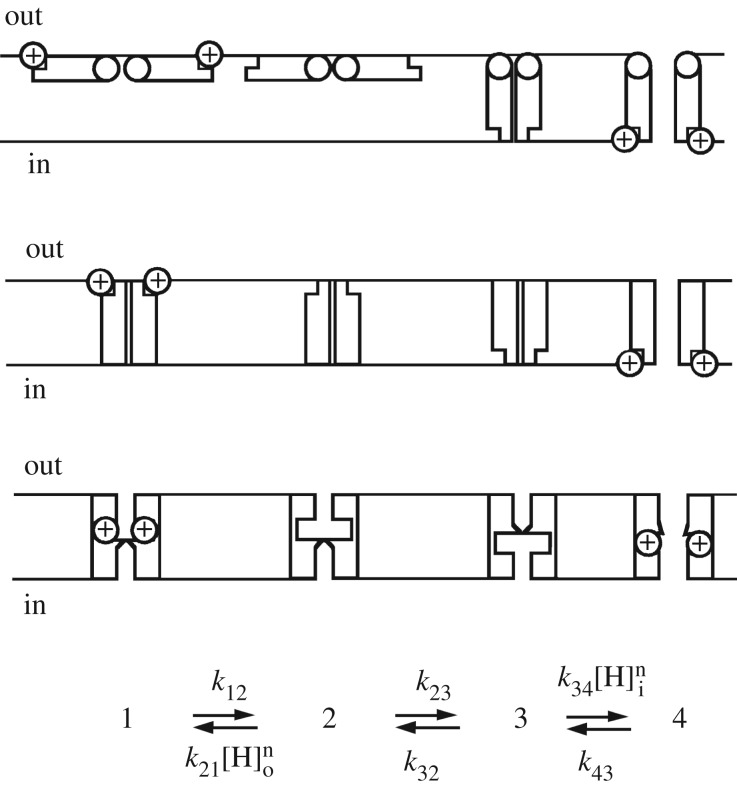
A four-state ‘butterfly’ model that explains the main features of ΔpH-dependent gating is shown with three possible physical representations. Channel opening occurs from left to right, with state 4 the only conducting state. Top row shows two ‘wings’ that cross the membrane exposing the sites to the opposite solution. The middle row depicts equivalent but distinct internal and external sites, of which only those on one side are accessible. The bottom row shows sites within the pore whose accessibility switches due to a subtle conformational change. Protonation from the external solution stabilizes the deepest closed (non-conducting) configuration (state 1). Deprotonation (state 1 → 2) is required before a conformational change switches the accessibility of the titratable groups to face inwardly (state 2 → 3). Finally, protonation from the inside (state 3 → 4) stabilizes the open channel (state 4). Because no single amino acid substitution abolishes ΔpH dependence [92], multiple groups are probably involved. Reprinted with permission from Cherny *et al.* [89] (Copyright © 1995 Rockefeller University Press).

Increasing pH_o_ or decreasing pH_i_ shifts the position of the *g*_H_–*V* relationship negatively by 40 mV per unit change in pH [89]. How does the channel sense pH, or more specifically, the pH gradient, ΔpH? Then, how does the channel transduce this perception into channel opening? Many enzymes are pH-sensitive, and they generally sense pH via protonatable groups. In a survey of 35 arbitrarily selected proteins, pH sensing was impaired by mutation of His in 20, Glu in 15, Asp in 7, Arg in 6, Lys in 6 and Gly in 3, and pH sensing frequently involved multiple amino acids [190]. It is difficult to envisage a pH-sensing mechanism that does not involve titratable amino acid side chains, although one exhaustive study of hH_V_1 found that mutation of several dozen individual titratable residues failed to eliminate or even attenuate ΔpH-dependent gating [92]. These authors concluded somewhat cryptically that ‘interactions between water molecules and S4 arginines may underlie coupling between voltage- and pH-gradient sensing’. The only explicit model to explain ΔpH-dependent gating postulated that one or more protonatable groups sense pH as shown in figure 5. This model accounts for the ΔpH dependence of gating by means of titratable groups on the channel that stabilize the closed or open conformation when protonated from the outside or the inside, respectively [89]. A crucial aspect of this model is a requirement for alternating access of the titratable groups; they are accessible to the external or internal solution but not at the same time, and accessibility changes occur only in the deprotonated condition. The voltage dependence may result from movement of charges through the membrane electrical field during the conformational change (states 2 ↔ 3) or from voltage-dependent binding or unbinding of protons to the titratable groups or both. The latter possibility was called a ‘proton well’ by Mitchell [191]. This model predicts the 40 mV shift of the *g*_H_–*V* relationship and qualitatively reproduces pH effects on gating kinetics [89]. Measurement of the pH dependence of gating transitions [89,192] provides a basis for refining such a model.

Recent indirect evidence indirectly supports a model for ΔpH-dependent gating that involves titratable sites. The WT hH_V_1 was shown to exhibit saturation of ΔpH dependence at pH_i_ or pH_o_ higher than 8.0 [193], which might be expected if the ambient pH were approaching the p*K*_a_ of one or more titratable groups. More surprisingly HtH_V_1, a proton channel from the snail *Helisoma trivolvis*, was identified whose *g*_H_–*V* relationship shifted only 20 mV or less when pH_i_ was varied, despite normal or even hyper-normal responses to changes in pH_o_ (greater than 50 mV per unit) [172]. One key difference between the sequences of snail and human H_V_1 was in the S2 and S3 intracellular linker. When His^168^ in human H_V_1 was replaced with glutamine, which occupies that position in HtH_V_1, the mutant human channel behaved like the snail, with greatly weakened pH_i_ sensitivity [194]! A shortened isoform of H_V_1 in human sperm, lacking the first 68 amino acids of the intracellular N terminus also has subnormal pH_i_ sensing [9]. Selective impairment in pH_i_ sensing is consistent with distinct internal and external pH sensors, as opposed to a centrally located sensor that samples pH on both sides of the membrane. Additional evidence that distinct external and internal sensors exist is that mutation of an unusual tryptophan in the hH_V_1 pore, Trp^207^, modifies pH_o_ sensing without affecting pH_i_ sensing [193].

### 5.3. The counter-charge model for ΔpH-dependent gating

**Figure 6.**
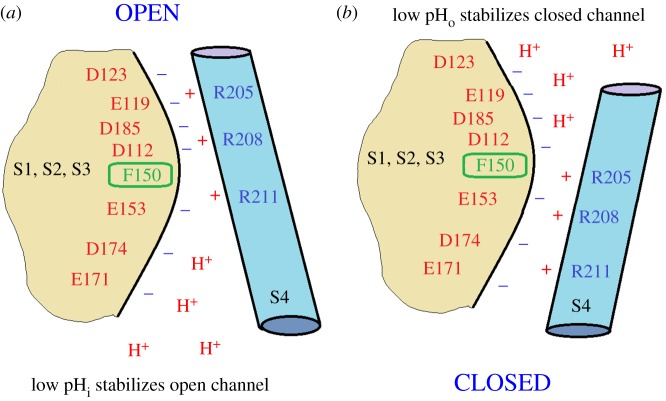
Cartoon illustrating the ‘counter-charge model’ for ΔpH-dependent gating of H_V_1. The premise is that both open and closed states of the channel are stabilized by interhelical electrostatic interactions. The main charged groups on the S4 helix, which is thought to move outwards during channel opening, are three Arg (blue). The S1–S3 segments have a number of acidic groups. Internal protons (low pH_i_) will tend to protonate acidic groups (*a*), preventing them from engaging in electrostatic interactions, thereby destabilizing the closed state and promoting opening. Conversely, external protons (low pH_o_) will protonate externally accessible acidic groups, destabilizing the open state and promoting channel closing. Positions of groups are highly schematic! The hydrophobic gasket that demarcates internal and external accessibility is depicted as a highly conserved Phe^150^ [143].

The identification of the gene for the voltage-gated proton channel H_V_1 [5,6] revealed its surprising homology with the VSD of K^+^, Na^+^ and Ca^2+^ channels (figure 1). Despite several distinct differences, for example, H_V_1 contains only three Arg residues in S4, the overall arrangement is similar. All VSDs have four transmembrane helices, with a series of cationic Arg or Lys in S4 that are thought to sense voltage, and several conserved acidic amino acids in S1–S3 that are thought to interact with the cationic residues to stabilize closed, open or intermediate states. The basic groups in S4 are thought to move outwards during channel opening, passing through a ‘hydrophobic gasket’ [91,100,195,196,] or ‘charge transfer centre’ [143] that includes an extremely highly conserved Phe (Phe^150^ in hH_V_1), which is the delimiter between inner and outer vestibules that access internal and external aqueous solutions, respectively. Furthermore, Cys scanning indicates that the general movement of S4 relative to the other domains (S1–S3) in proton channels [85] is qualitatively similar to the movement that occurs in other voltage-gated ion channels [63,133–135]. Thus, one or more Arg residues are accessible to the internal solution in the closed state, but move outwards past a short constriction (depicted in figure 6 as the highly conserved Phe^150^), to become accessible to the external solution in the open state. We assume that the Arg in S4 contribute to the voltage dependence of gating [178], as they do in other ion channels [130,131,140,152,154]. The high p*K*_a_ of Arg means that it will remain positively charged under almost all conditions, a desirable property for a voltage-sensing element. One of the unique features of voltage-gated proton channels is that their voltage-dependent gating is strictly regulated by the pH gradient, ΔpH. Specifically, increasing pH_o_ or decreasing pH_i_ by one unit shifts the *g*_H_–*V* relationship by −40 mV [89]. This regulation results in the proton channel opening only when the electrochemical gradient is outwards (figure 4), such that opening will result in acid extrusion from cells [89]. This property is observed in all voltage-gated proton channels identified to date, and is crucial to the physiological roles of this channel [60,68].

The model in figure 6 illustrates a hypothetical mechanism for the ΔpH dependence of gating. In this model, electrostatic interactions between the Arg in S4 and acidic residues in other transmembrane segments regulate the ΔpH dependence of gating. This kind of charge–pair interaction has been proposed to occur within the VSD during gating of other voltage-gated ion channels, stabilizing closed or open states [146,147,197]. An additional twist added to this strategy by H_V_1 is that charge–pair interactions can be inhibited by protonating the acidic member of the pair. In this way, pH naturally exerts the effects that are predicted more generally by the model in figure 5. Protonation of acidic groups that are accessible to the internal solution in the closed state destabilizes their interaction with Arg, promoting channel opening. Conversely, protonation of groups that are externally accessible in the open state destabilizes the open state by eliminating their interaction with Arg residues, thus promoting channel closing. Several acidic residues unique to proton channels (i.e. lacking homology with the VSD of other channels), such as Asp^112^ and Asp^185^ [82], may contribute to this mechanism, in addition to acidic residues homologous to those thought to interact electrostatically with S4 Arg residues in K^+^ channels [146,147,198–200]. Given that no single point mutation abolishes ΔpH dependence [82,92], the mechanism that produces the ΔpH-dependent gating crucial to proton channel physiology is evidently robust and incorporates redundancy.

Strong experimental support for this type of model exists [82,92]. Neutralizing acidic residues that are internally accessible and interact with Arg preferentially in the closed state should destabilize the closed state and promote the open state. In other words, neutral mutants of internal acidic amino acids should shift the *g*_H_–*V* relationship negatively. Two key residues whose neutralization promotes the open state are Asp^174^ and Glu^153^ [82,92,96,201]. Conversely, neutralizing acidic residues that are externally accessible and interact with Arg preferentially in the open state should destabilize the open state and promote channel closing. Replacing acidic amino acids with neutral ones should shift the *g*_H_–*V* relationship positively, and this has been reported for Asp^112^, Asp^123^ and Asp^185^ [82,92,96].

Despite the simplicity and intuitive appeal of the counter-charge model, an explicit quantitative model has not been published, and other types of models can be envisaged. Understanding the mechanism of ΔpH-dependent gating of H_V_1 remains an elusive pimpernel.

## 6. Physiological modulators of gating

Several physiological molecules modulate H_V_1 gating, in each case increasing the sensitivity to voltage as well as altering the kinetics of the response. The best characterized response is a constellation of four profound changes called the ‘enhanced gating mode’, which occurs in phagocytes during the ‘respiratory burst’ when phagocytosed bacteria are killed or agonists like chemotactic peptides are applied [57,189,202]. The proton current increases, activation (channel opening) becomes much faster, deactivation (channel closing) much slower and the *g*_H_–*V* relationship shifts negatively by 40 mV [57,60,68,202,203]. In most cases, the signalling pathway involves protein kinase C (PKC) [56,60,68,204–206], which phosphorylates the hH_V_1 molecule at Thr^29^ in the intracellular N terminus [8,55]. Another type of gating enhancer is arachidonic acid [207] and other unsaturated long-chain fatty acids [42,208]. Arachidonic acid can increase H^+^ currents directly [206–212], but can also act indirectly by activating PKC [206]. The actual physical mechanism by which gating enhancers enhance gating is unknown. The PKC phosphorylation site Thr^29^, for example, is located in the mostly disordered intracellular N terminal region, and how it manages to influence gating can only be speculated.

## 7. Summary

Given the uncertainties in interpreting data and the inaccessibility of the events and structures responsible for gating and conduction in hH_V_1, what conclusions can we draw? To some extent, gating and conduction are not such clearly separable processes as they are in other channels. Protons both carry current and also tightly regulate when the channel will open or close. Gating at minimum requires rearrangement between conformations that permit or prevent selective H^+^ conduction. That gating is regulated by both voltage and pH constrains possible mechanisms. Although little molecular movement is required to effect gating by *a priori* considerations, many types of evidence support some movement occurring, especially of the S4 helix. The dynamic nature of hH_V_1 revealed by EPR means that there is extensive motion [91], but the nature of the motion is unspecified. That the selectivity filter retains function when repositioned from position 112 to 116 in the S1 helix (WT Asp^112^ to V116D) means that there is some leeway in creating an open and H^+^-selective conducting state, but the fact that moving Asp to other locations failed to produce H^+^ current means that H^+^-selective conduction has fairly stringent requirements [95]. That many point mutations cause loss of selectivity or abolish function altogether [82] indicates that there are a number of places in the H_V_1 molecule where arbitrary changes are not allowed. Evidently, it is easier to impair function than to explain it. The more exotic mechanism of ΔpH-dependent gating most probably involves titratable sites, but if so, these must exhibit redundancy, because ΔpH dependence is not eliminated by single point mutations [82,92].

## Endnote

1 Amino acids in proteins are numbered starting from the N terminus. Because there are differences in the primary sequences in different species, the numbering of equivalent positions differs. The first Arg in S4 in human H_V_1 is at position 205, but in mouse it is 201.

## Data accessibility

This article has no additional data.
